# A47 MICROBIAL FUNCTIONS AS BIOMARKERS OF PRO-INFLAMMATORY RESPONSE TO SELECT DIETARY FIBERS IN IBD

**DOI:** 10.1093/jcag/gwab049.046

**Published:** 2022-02-21

**Authors:** H Armstrong, M Bording-Jorgensen, R Valcheva, Z Zhang, J Jovel, A Petrova, M W Carroll, H Q Huynh, L A Dieleman, E Wine

**Affiliations:** 1 University of Alberta, Edmonton, AB, Canada; 2 Pediatrics, University of Alberta Faculty of Medicine and Dentistry, Edmonton, AB, Canada; 4 Jimei University, Xiamen, Fujian, China; 5 Pediatric Gastroenterology, Univeristy of Alberta, Edmonton, AB, Canada; 6 Pediatrics, University of alberta, Edmonton, AB, Canada; 7 Medicine, University of Alberta, Edmonton, AB, Canada

## Abstract

**Background:**

Dietary fibers are not digested in the bowel; they are fermented by microbes, typically promoting gut health. However, IBD patients experience sensitivity to consumption of fibers. Our previous findings offered the first mechanistic evidence demonstrating that unfermented dietary β-fructans (inulin and FOS) can induce pro-inflammatory cytokines in a subset of pediatric IBD colonic biopsies cultured *ex vivo*, and in the SYNERGY-1 (β-fructan) clinical study of adult remission UC patients. Incubating FOS with whole-microbiota intestinal washes from non-IBD or remission IBD patients improved fermentation and reduced pro-inflammatory responses, but not from patients with active disease. Fibre-induced immune responses correlated with microbe functions, luminal metabolites, and fibre avoidance.

**Aims:**

Here we aimed to expand on our findings and define the role of microbial functions in mediating host response to β-fructans.

**Methods:**

Colonic biopsies cultured *ex vivo* and cell lines *in vitro* were incubated with FOS (5g/L), or fermentation supernatants (24hr anaerobic fermentation). Immune responses (cytokine secretion [ELISA/MSD] and expression [qPCR]) were assessed. Taxonomic classification of microbial fermentation cultures was conducted with Kraken2 and metabolic profiling by HUMAnN2. HPLC and gas chromatography volatile fatty acid (CG-VFA) analysis were used to identify concentrations of remaining fibre and SCFAs following anaerobic fermentation.

**Results:**

7 microbial enzymes were identified to be predictive of cytokine (IL-1β, IL23, IL-5, IL-8, MIP-1α) secretion in *ex vivo* colonic biopsies from pediatric Crohn disease (CD; n=38), ulcerative colitis (UC; n=20), and non-IBD (n=21) patients, in response to β-fructans; their use as biomarkers of response was determined in patient stool from the SYNERGY-1 clinical study cohort. Fermentation of FOS by whole-microbe intestinal washes from only non-IBD or remission IBD patients reduced cytokine secretion, and our findings demonstrate that this was due to a combination of reduction of β-fructan present and production of a precise combination of anti-inflammatory SCFAs.

**Conclusions:**

Our findings suggest that intolerance and avoidance of fibers in select IBD patients is associated with the inability to ferment these fibers, mediated by altered microbial functions (enzymes), leading to worsened inflammation. Data indicate that gut microbial function, not composition, predicts patient pro-inflammatory response to β-fructans, supporting our hypothesis that overall community function impacts fibre fermentation and affects associated pro-inflammatory effects. Our work highlights select disease state scenarios in which administration of fermentable fibers should be avoided and tailored dietary interventions considered.

**Funding Agencies:**

CIHRWeston Foundation, Mitacs

